# P09-04 Sedentary time measured by GT3X+ accelerometry and its variation with grade level and gender among children and adolescents in Morocco

**DOI:** 10.1093/eurpub/ckac095.134

**Published:** 2022-08-29

**Authors:** Asmaa El Hamdouchi, Imane El Harchaoui, Kaoutar Benjeddou, Imane El Menchawy, Naima Safsaf, Naima Saeid, Khalid El Kari, Mohammed Elmzibri, Issad Baddou, Hassan Aguenaou

**Affiliations:** Unité Mixte de Recherche Nutrition et Alimentation CNESTEN- Ibn Tofail University (URAC39), Regional Designated Center of Nutrition Associated with AFRA/IAEA. Morocco., Rabat, Morocco

**Keywords:** sedentary. GT3X+. weekday. weekend. children. adolescent

## Abstract

**Background:**

Sedentary behavior (SB) in children is related to different health outcomes such as overweight and cardio-metabolic diseases. These negative effects have been widely supported by evidence. However, no data on sedentary time (ST) among Moroccan children has been available, yet. Therefore, the present study examined gender and grade differences in objectively measured sedentary behavior in a sample of Moroccan primary school children and adolescents.

**Methods:**

In total, 172 Moroccan children/adolescents aged between 8 to 14 years old (mean age = 10.92 ± 1.55 years; 49.4% were boys) completed the survey. School grade, gender, height, and weight were collected by questionnaires and ST objectively measured using a tri-axial accelerometer (GTX3+). Study required at least 3 valid weekdays and 1 weekend day with? 600 min/day total wear time. Two-way analysis of covariance and logistic regression analyses, adjusted for BMI z-score and accelerometer wear time, were used to examine gender and grade differences in ST.

**Results:**

Mean time spent in SB was 535.93 ± 87.15 min/day or ∼ 62.94% of the average daily accelerometer wear time of 851.45 ±51.35.min/day with statistical differences between weekend and week days (471.357 ± 127.73 minutes/day vs. 559.7661± 90.75 minutes/day; p > 0.001). Adolescents (11-14y) were more involved in sitting tasks when compared to the early grades (8-10y). 550.011±88.827 vs. 521.845±83.602 respectively; p > 0.001.

**Conclusion:**

ST increases between ages 11 and 14 years. On week days children and adolescents spent sitting longer than at weekends. Girls and adolescents were identified as potential risk groups. This report on ST presents valuable information for designing and implementing interventions to decrease time spent in SB among children during class time.

**Acknowledgements**

This study was performed with the support of the International Atomic Energy Agency (CRP E4.30.24; RAF 6042).

